# Biomechanical characteristics of the lower extremities during running in male long-distance runners with a history of medial tibial stress syndrome: a case control study

**DOI:** 10.1186/s12891-023-06216-0

**Published:** 2023-02-07

**Authors:** Takehiro Ohmi, Junya Aizawa, Kenji Hirohata, Shunsuke Ohji, Sho Mitomo, Toshiyuki Ohara, Kazuyoshi Yagishita

**Affiliations:** 1 Clinical Center for Sports Medicine and Sports Dentistry, Tokyo Medical and Dental University, 1-5-45 Yushima, Tokyo, Bunkyo-Ku 113-8519 Japan; 2 Department of Physical Therapy, Faculty of Health Science, Juntendo University, Tokyo, Japan

**Keywords:** Kinetics, Medial tibial stress syndrome, Biomechanical phenomena, Running

## Abstract

**Background:**

Medial tibial stress syndrome (MTSS) is a running-related injury of the lower extremities. After returning to competition, there are often recurring episodes of MTSS. Therefore, it is important to prevent the onset and recurrence of MTSS among long-distance runners. This case–control study aimed to compare the kinematics and kinetics of runners with and without previous MTSS during running to clarify the biomechanical characteristics of the lower extremity of runners with previous MTSS.

**Methods:**

Thirteen male long-distance runners aged over 18 years and asymptomatic at the time of measurement were divided into an MTSS group and a non-MTSS group based on their history of MTSS as reported in a questionnaire. The kinetics and kinematics of running were analyzed when participants ran at a speed of 2.0 ± 0.2 m/s by a three-dimensional motion analysis system and two force plates. Data regarding the joint angles, moments, and powers of the ankle, knee, and hip during the stance phase while running were extracted and compared between the two groups using the Mann–Whitney U test.

**Results:**

Of the 13 participants, 5 and 8 were included in the MTSS (10 legs) and non-MTSS (16 legs) groups, respectively. The ankle maximum eversion moment was significantly larger in the MTSS group than in the non-MTSS group (*p* = 0.04). There were no significant differences in other parameters.

**Conclusions:**

This study found that the ankle maximum eversion moment during the stance phase of running was larger in the MTSS group than in the non-MTSS group. Even after the disappearance of the symptoms of MTSS, the running biomechanics of participants with previous MTSS differed from those of participants without previous MTSS.

## Background

Many adult long-distance runners (19–92%) have experienced a running-related injury of the lower extremities [1]. Medial tibial stress syndrome (MTSS) accounts for 5–10% of runners [2, 3]. This injuries occurs at 0.01 per 1000 km in elite runners [4]. Runners may require prolonged periods of rest and cessation of running, ranging from 2 to 6 weeks on average [2]. After returning to competition, MTSS often recurs [3]. MTSS incidence in runners with an MTSS history in one season is about twice that of runners without such a history [4, 5]. Therefore, prevention of the onset and recurrence of MTSS in long-distance runners is important. Previous findings demonstrate the multifactorial development of MTSS, involving passive range of motion, muscle strength, plantar pressure distributions, and both proximal and distal kinematics [6]. A study involving 146 college athletes showed that 87% had a history of MTSS [7]. A history of MTSS is a risk factor for MTSS recurrence [2, 8, 9].

Compared to runners with no previous injuries, including MTSS or stress fractures, runners with previous injuries have biomechanical characteristics including greater hip flexion or pelvic tilt angles in the frontal plane and lesser knee flexion angles in the stance phase of running [10, 11]. Therefore, even after MTSS symptoms have disappeared, the biomechanics affecting recurrence of MTSS may remain.

A study that analyzed running in individuals with a previous running injury looked at ground reaction forces and joint angles, but not joint moments or power [10, 11]. These kinematic variables are important for estimating the load on muscles and joints during motion. Previous reports [10, 11] have assessed multiple running disorders of the shanks and thighs. The kinematic factors of running injuries such as MTSS differ from those of injuries of the thigh. However, no study has analyzed MTSS-related kinetics data during running.

We aimed to compare the kinematics and kinetics of runners with and without previous MTSS during running to clarify the biomechanical characteristics of runners with previous MTSS. Excessive traction stress to the soleus fascia and tibial periosteum during running is involved in MTSS onset [12, 13]. Thus, we hypothesized that runners with and without previous MTSS have different eversion angles and moments, both of which are associated with increased traction stress on the soleus fascia and tibial periosteum [12].

## Methods

### Study design and oversight

This was a case-control study that followed the guidelines of the 1975 Declaration of Helsinki. Our study were performed in accordance with relevant guidelines and regulations. Ethical approval was granted by our institutional review board (approval number, M 2000-2069), and all participants provided written informed consent before participation.

### Participants

Male long-distance runners aged 18–30 years were included who (i) specialized in long-distance running competitions at the university level or higher; (ii) participated in training ≥ 3 times per week; and (iii) reported no pain in their lower extremities at the time of the assessment. Participants who had not participated in practice sessions for more than one week within the last six months due to injury or illness were excluded. Thirteen participants (age, 24.1 ± 1.8 years; height, 170.1 ± 5.7 cm; weight, 55.3 ± 4.2 kg; body mass index, 19.1 ± 0.6 kg/m^2^) who met the criteria were included. A leg was counted as one sample [14–16] in investigating the kinetics and kinematics of a leg with an MTSS history.

### Questionnaire

Participants described their mileage and MTSS history in the self-administered questionnaire. The MTSS history was defined according to a previous diagnosis of MTSS by a doctor who ruled out a tibial stress fracture or exertional compartment syndrome via diagnostic imaging. The running mileage was defined as the total distance travelled by the participant during practice or in competitions in the last week before the assessment [11].

### Measurement of motion

All participants wore identical athletic attire comprising spandex shirts, shorts, and shoes without air cushions (step101, Lucky Bell, Kobe, Japan). Participants were assessed using a three-dimensional motion analysis system (Motive; Acuity Inc., Tokyo, Japan) with eight cameras operating at a sampling rate of 100 Hz and two force plates (TF-406; Tec Gihan Co, Kyoto, Japan) at a sampling rate of 1,000 Hz. Sixteen infrared reflective markers (9 mm spheres) were attached to anatomical landmarks using the Plug-in-Gait model (Oxford Metrics LTD, Oxford, United Kingdom). Markers were placed over both anterior superior iliac spines and the posterior superior iliac spine, lateral thigh, lateral condyles of the thigh, lateral shank, lateral malleolus, posterior calcaneus, and second metatarsal heads. The motor task was running on a track at a speed of 2.0 ± 0.2 m/s for approximately 10 m [17, 18]. Before the running task, participants warmed up by stretching or running for 5 min and practicing running at a predetermined speed. A successful measurement occurred when the patient’s entire foot was placed on the force plate surface.

### Analysis

The three-dimensional data were smoothed by a second-order Butterworth-type low-pass filter with a cut-off frequency of 10 Hz and imported to analysis software (SKYCOM; Acuity Inc, Tokyo, Japan). A lower extremity model of the pelvis, thighs, shanks, and foot segments was created using this software. Additionally, using this model, the kinematics and kinetics of the hip, knee, and ankle on the sagittal and frontal planes were analyzed (Table 1). The eversion or inversion moment is the external moment when a foot segment is applied outward or inward to the direction of progression. Joint moments were calculated as external joint moments and were normalized to the body weight (Nm/kg). Furthermore, joint powers were normalized to body weight (W/kg).

**Table 1:** Study Parameters

Joint	Kinetic/kinematic variable	Orientation
Ankle	Angle	Planter flexion/Dorsi flexion eversion/inversion
	Moment	Planter flexion/Dorsi flexion eversion/inversion
	Power	Sagittal plane Frontal plane
Knee	Angle	Flexion/Extension Varus/Valgus
	Moment	Flexion/Extension Varus/Valgus
	Power	Sagittal plane Frontal plane
Hip	Angle	Flexion/Extension Adduction/Abduction
	Moment	Flexion/Extension Adduction/Abduction
	Power	Sagittal plane Frontal plane

### Grouping according to MTSS history

The participants who reported an MTSS history were asked to provide more details. The survey asked, "In which leg did you previously experience MTSS?" and "How many times did you experience MTSS?".

Legs reported to have previous MTSS were included in the MTSS group, and those without reported MTSS history were included in the non-MTSS group.

### Statistical analysis

Participant demographics and questionnaire results were descriptively summarized. Data normality was confirmed using the Shapiro–Wilk test. An unpaired t-test was used to compare normally distributed data between the groups. Finally, the Mann–Whitney U test was used to compare the non-normally distributed data between groups, and the effect size (d) or (r) was calculated. All statistical analyses were performed using SPSS version 23.0 (IBM Corp., Armonk, NY, USA). All statistical significance was set at *p* < 0.05.

An a priori sample size calculation was conducted using G* Power software 3.1.9.4 [19]. Based on a previous study [6] that analyzed the kinematics of the ankle, the minimum number of participants was calculated with an α value of 0.05 and β of 0.8, and 12 paired samples in each group were indicated.

## Results

### Demographic data

Of the 13 participants, 5 had a history of MTSS (10 legs; MTSS group), and 8 did not (16 legs; non-MTSS group). All five MTSS participants had a history of MTSS in both legs. In the motion analysis, 10 MTSS legs and 16 non-MTSS legs (total: 26 legs) were analyzed. No participants had previous tibial stress fractures, and no significant differences were found in participant demographics between the two groups (Table 2). In the MTSS group, the median time of the previous MTSS onset was 1.5 [4.0] years (median [interquartile range]).

**Table 2:** Participant Demographics

	**MTSS group (*n* = 5)**	**non-MTSS group (*n* = 8)**	***p*-value**
Age (years)	23.600 ± 2.408	24.200 ± 1.924	0.556
Height (cm)	171.700 ± 5.805	167.520 ± 7.222	0.466
Weight (kg)	55.700 ± 4.550	53.400 ± 4.827	0.831
BMI (kg/m^2^)	18.874 ± 0.827	18.998 ± 0.272	0.294
Running mileage (km)	154.000 ± 18.166	142.000 ± 4.472	0.109

Mean ± standard deviation

*Abbreviations*: *BMI* Body mass index, *MTSS* Medial tibial stress syndrome

### Biomechanical data

**Fig. 1:**
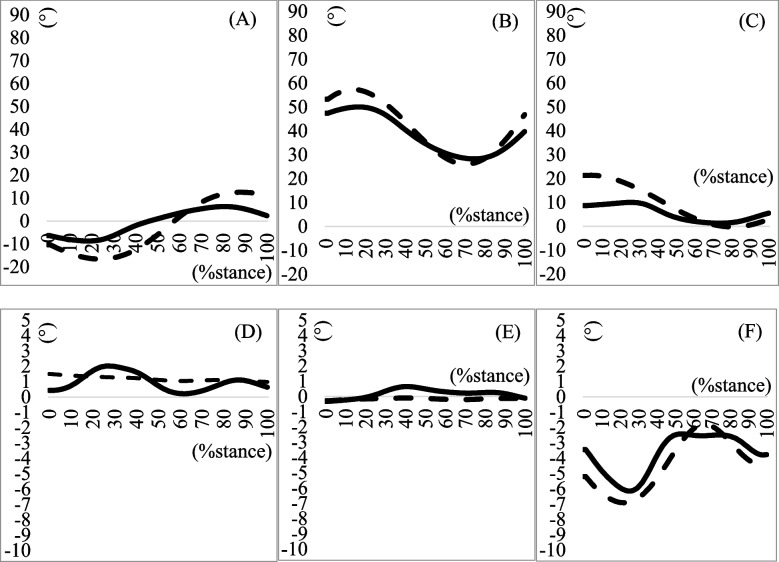
Joint angle during running in the stance phase. The solid line and dashed line represent the MTSS group and Non-MTSS group, respectively. **A** Ankle joint dorsi ( +)/planter flexion angle (-); **B** Knee joint flexion ( +)/extension (-) angle; **C** Hip joint flexion ( +)/extension (-) angle; **D** Ankle joint eversion ( +)/inversion (-) angle; **E** Knee joint varus ( +)/valgus (-) angle; **F** Hip joint adduction ( +)/abduction (-) angle. MTSS, medial tibial stress syndrome

**Fig. 2:**
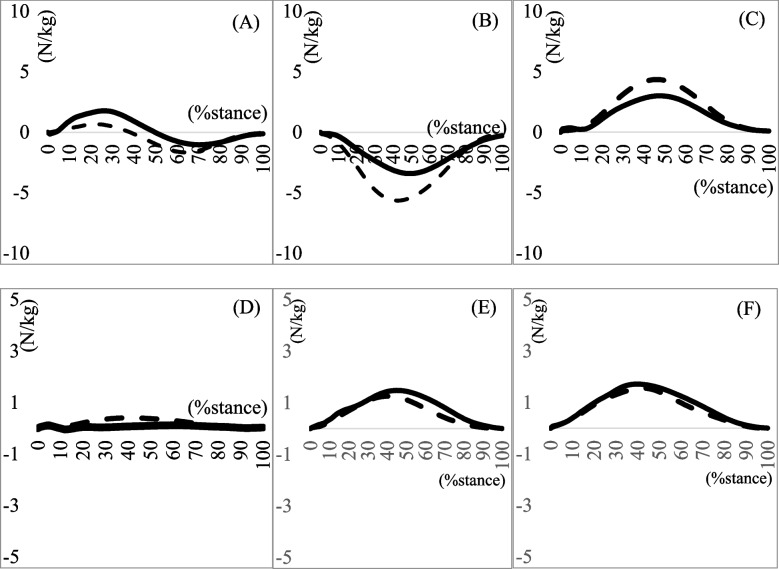
Joint moments during running in the stance phase. The solid line and dashed line represent the MTSS group and Non-MTSS group, respectively. **A** Ankle joint dorsi ( +)/planter (-) flexion moment; **B** Knee joint flexion ( +)/extension (-) moment; **C** Hip joint flexion ( +)/extension (-) moment; **D** Ankle joint eversion ( +)/inversion (-) moment; **E** Knee joint varus ( +)/valgus (-) moment; **F** Hip joint adduction ( +)/abduction (-) moment.MTSS, medial tibial stress syndrome

**Fig. 3:**
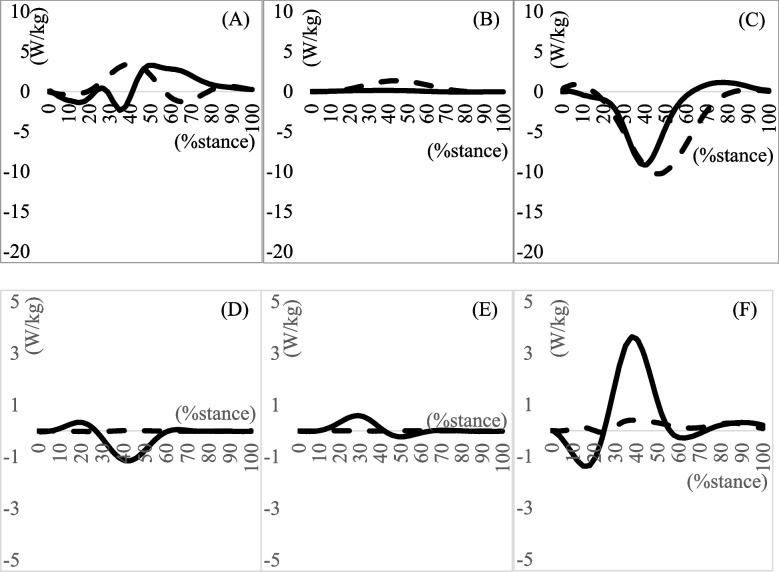
Joint power during running in the stance phase. The solid line and dashed line represent the MTSS group and Non-MTSS group, respectively. **A** Ankle joint power on the sagittal plane; **B** Knee joint power on the sagittal plane; **C** Hip joint power on the sagittal plane; **D** Ankle joint power on the frontal plane; **E** Knee joint power on the frontal plane; **F** Hip joint power on the frontal plane. MTSS, medial tibial stress syndrome

Biomechanical data of the lower extremities are shown in (Tables 3, 4 and 5), and (Figs. 1, 2 and 3). No significant difference was found in the maximum eversion angle between the groups (*p* = 0.77). However, the maximum eversion moment was significantly larger in the MTSS group than in the non-MTSS group (*p* = 0.03) (Table 3). No differences were found in the knee and hip biomechanical data during the stance phase of running (Tables 4 and 5).

**Table 3:** Kinematics and Kinetics of the Ankle During Stance Phase of Running

	**MTSS group** **(10 legs)**	**non-MTSS group** **(16 legs)**	***p*-value**	**Effect size**
Maximum dorsi flexion angle (°)	15.327 ± 7.347	14.890 ± 7.670	0.890	0.060
Maximum plantar flexion angle (°)	20.458 (14.160)	23.601 (17.275)	0.879	0.070
Maximum dorsi flexion moment (Nm/kg)	1.202 (3.230)	1.756 (1.101)	0.267	0.730
Maximum plantar flexion moment (Nm/kg)	2.446 (2.823)	2.213 (0.799)	0.796	− 0.020
Maximum eversion angle (°)	1.512 (0.905)	2.505 (2.946)	0.279	0.480
Maximum inversion angle (°)	0.303 (0.813)	0.214 (1.110)	0.765	0.120
Inversion–eversion excursion angle (°)	1.403 ± 0.717	2.597 ± 1.514	0.071	0.850
Maximum inversion moment (Nm/kg)	0.401 ± 0.404	0.207 ± 0.313	0.279	0.550
Maximum eversion moment (Nm/kg) *	0.405 ± 0.444	0.100 ± 0.134	0.03	1.101
Maximum generated power on the sagittal plane (W/kg)	7.647 (9.812)	9.501 (8.134)	0.547	0.170
Maximum absorptive power on the sagittal plane (W/kg)	5.404 (9.512)	2.207 (3.835)	0.990	0.000
Maximum generated power on the frontal plane (W/kg)	0.044 ± 0.038	0.100 ± 0.094	0.107	0.730
Maximum absorptive power on the frontal plane (W/kg)	0.073 ± 0.081	0.148 ± 0.154	0.188	0.580

Mean ± standard deviation, median (interquartile range); *Significance at *p* < 0.05

*Abbreviations*: *MTSS* Medial tibial stress syndrome

**Table 4:** Running in stance phase kinematics and kinetics of the knee

	**MTSS group**	**non-MTSS group**	***P*-value**	**Effect size**
Maximum flexion angle in degrees	44.463 ± 4.145	44.981 ± 3.364	0.730	0.140
Maximum extension angle in degrees	− 13.235 (8.312)	− 21.475 (6.601)	0.083	0.790
Maximum flexion moment in Nm/kg	− 0.123 (0.458)	0.042 (0.173)	0.346	0.491
Maximum extension moment in Nm/kg	4.581 (2.100)	4.203 (4.946)	0.222	0.470
Maximum varus angle in degrees	0.013 (0.425)	0.019 (0.471)	0.375	0.480
Maximum valgus angle in degrees	0.043 (0.449)	0.018 (0.126)	0.376	0.410
Maximum varus moment in Nm/kg	1.517 ± 0.915	1.057 ± 0.714	0.165	0.630
Maximum valgus moment in Nm/kg	− 0.154 (0.102)	0.017 (0.189)	0.734	0.210
Maximum generated power on the sagittal plane (W/kg)	16.234 (20.222)	15.903 (57.000)	0.353	0.250
Maximum absorptive power on the sagittal plane (W/kg)	5.987 (13.102)	6.149 (6.432)	0.193	0.770
Maximum generated power on the frontal plane (W/kg)	0.040 (0.090)	0.012 (0.050)	0.224	-0.210
Maximum absorptive power on the frontal plane (W/kg)	0.025 (0.080)	0.014 (0.030)	0.516	-0.120

Mean ± standard deviation, median (interquartile range)

*Abbreviations*: *MTSS* Medial tibial stress syndrome

**Table 5:** Running in stance phase kinematics and kinetics of the hip

	**MTSS group**	**non-MTSS group**	***P*-value**	**Effect size**
Maximum flexion angle in degrees	19.717 ± 12.588	24.285 ± 7.491	0.323	0.460
Maximum extension angle in degrees	11.017 (11.350)	7.958 (21.160)	0.215	0.20
Maximum flexion moment in Nm/kg	3.088 ± 2.596	4.390 ± 2.632	0.243	0.500
Maximum extension moment in Nm/kg	0.082 ± 0.302	0.141 ± 0.232	0.607	0.220
Maximum abduction angle in degrees	0.638 ± 5.430	0.421 ± 2.057	0.569	0.060
Maximum adduction angle in degrees	7.422 (7.250)	9.439 (6.910)	0.585	0.270
Maximum abduction moment in Nm/kg	1.911 ± 1.108	1.682 ± 0.602	0.520	0.270
Maximum adduction moment in Nm/kg	0.025 (0.180)	0.007 (0.100)	0.709	0.300
Maximum generated power on the sagittal plane (W/kg)	3.911 (5.650)	1.171 (1.670)	0.084	1.180
Maximum absorptive power on the sagittal plane (W/kg)	− 5.6 70(14.440)	− 7.007 (11.600)	0.546	0.500
Maximum generated power on the frontal plane (W/kg)	1.844 ± 0.947	2.039 ± 0.603	0.854	0.260
Maximum absorptive power on the frontal plane (W/kg)	1.350 ± 0.686	0.780 ± 0.572	0.048	0.920

Mean ± standard deviation, median (interquartile range)

*Abbreviations*: *MTSS* Medial tibial stress syndrome

## Discussion

This study found that the maximum eversion moment during the stance phase of running was larger in runners with an MTSS history than in those without an MTSS history. This finding partially supports the hypothesis of the study.

The maximum eversion moment in the MTSS group was significantly larger than that in the non-MTSS group (Table 3). Since no previous research has analyzed the joint moment and power in runners with an MTSS history, it is difficult to compare our results with previous reports. Therefore, our results will be discussed based on the MTSS etiology. MTSS is caused by a bone stress reaction to the impact of walking and running in the tibial cortex [18, 20]. Elongational stress on the soleus, flexor digitorum longus, and tibialis posterior muscles increases strain on the tibial fascia [18, 20]. Traction stress on the soleus and posterior tibial muscles is greater during movement and induces a large external eversion moment. In this study, traction stress on the soleus and posterior tibial muscles appeared relatively large in the MTSS group due to the large maximum eversion moment during running. Although no specific joint motion was observed in the MTSS group, they may not control the eversion moment.

One reason for the difference in the eversion moment between the two groups may be variations in the trajectory of the center of pressure (COP) during running. The stance phase COP during the gait, in those without MTSS, passed from the heel to the lateral forefoot and then to the medial toes. In runners who developed MTSS, the COP was transmitted directly from the heel to the medial toes [20, 21]. In the MTSS group, the COP might have passed through the medial sole, shortening the eversion moment lever arm. Since the COP trajectory during running was not analyzed in this study, future studies should explore a detailed kinematic analysis of the foot pressure distribution during running.

No significant intergroup differences were found in the maximal eversion and inversion angles and eversion-inversion angle excursion. The foot abduction, eversion, is considered to positively correlate with eversion in the subtalar because it is one of the pronation-in-subtalar-movement components [22, 23]. The soleus muscle and its fascia are more stretched during calcaneal inversion than during pronation [12]. However, our results do not support this hypothesis. Previous studies reported that athletes and military personnel with MTSS had a greater hindfoot eversion angle during running than those without MTSS [7, 24, 25]. A possible explanation for our results not supporting the hypothesis may be due to differences in foot definitions during the motion analysis and participant demographics. We defined the foot based on markers for the calcaneus, lateral malleolus of the fibula, and the second metatarsal head. Therefore, the forefoot and hindfoot were considered a single segment. However, another study defined the forefoot and hindfoot as separate segments [7]. They targeted athletes from several sports (for example, short-track running, tennis, and basketball). In contrast, our study targeted only long-distance runners. The onset mechanism of MTSS may differ between the above-mentioned sports and long-distance running.

The maximum ankle dorsiflexion angle did not differ significantly between the two groups. Okunuki [7] reported no significant differences in the foot dorsiflexion angle between MTSS and control groups. Interestingly, our results support this finding. The maximum dorsiflexion angle of the ankle during running appeared unrelated to the history or the symptoms of MTSS.

We found no significant intergroup differences in the maximum knee and hip angles during running. However, it has been reported that runners who experienced injuries demonstrated lesser knee flexion and greater hip adduction angles in the early stance phase than those without such a history [11, 26]. Other studies reported that runners with a history of bone stress injury (tibial stress fractures and shin splints) had greater hip flexion angles than runners without such a history [10, 26]. Our results did not support these findings, possibly because running injuries other than MTSS were included as target injuries in two of these studies [10, 26]; in the third study, participants were grouped according to the presence or absence of medial tibial pain [11]. Another reason may be that the analyzed running phases differ [22]. However, no study has analyzed joint moment and power in runners with MTSS or a history of running-related injuries. Our data suggest that knee and hip joint angles, moments, and powers during running were unrelated to the MTSS history.

In this study, a statistical difference was found in the ankle eversion moment and hip maximum absorptive power on the frontal plane; however, no difference was found in the other angles and moments on the frontal plane. Therefore, it is possible that the trunk movement, including the head and arms, contributed to the ankle eversion moment. However, this study did not measure it; therefore, it was difficult to prove.

Our biomechanical data partially describe the kinetics and kinematics in runners with previous MTSS. Therefore, the foot kinetics and kinematics in runners with an MTSS history should be evaluated to prevent recurrence after MTSS symptoms disappear.

This study had some limitations. First, this study’s results may not apply to female runners or runners of other categories because the participants were all male long-distance runners at the university level or higher. Second, since this was a case–control study, it is unclear whether the differences in the eversion moment caused MTSS or resulted from MTSS. Moreover, we cannot show that participants in the MTSS group acquired pain-avoidance movements from a previous MTSS, as the MTSS onset in the MTSS group was approximately 1.5 years before the study initiation. Therefore, future follow-up studies and prospective trials are warranted. Third, all MTSS participants had a bilateral MTSS history, and these results may not apply to individuals with a unilateral MTSS history. Additionally, this study did not analyze the difference between the dominant and non-dominant legs. This was because the dominant and non-dominant legs during running did not differ [27]. Fourth, parameters where *p* > 0.05 had a large effect size; therefore, type II errors were possible. This study had a small sample size; therefore, it might also have had a type II error. A post hoc power analysis revealed that, with an alpha value of 0.05 and effect size 1.10 (Maximum ankle joint eversion moment), this study had a power of 84.3% for the difference between the two groups. Lastly, kinetics and kinematics vary during fast motion [17, 28]. Although we set the running speed to 2.0 ± 0.2 m/s, our results may not be applicable at higher running speeds. Therefore, biomechanical data at different running speeds require further investigation.

## Conclusions

This study compared lower extremity kinetic and kinematic variables during running between a population of young adult male runners with or without an MTSS history. Our findings revealed that foot kinematics during the stance phase of running differed between individuals with and without a history of MTSS. Therefore, our findings suggest that running kinematics in runners with and without an MTSS history were different even after symptom disappearance.

## Data Availability

All data generated or analyzed during this study are included in this article.

## Abbreviations

COP: Center of pressure
MTSS: Medial tibial stress syndrome
